# Poly[[diaqua­bis­(μ_2_-4,4′-bipyrid­yl)cobalt(II)] dinitrate tetra­hydrate]

**DOI:** 10.1107/S1600536813005230

**Published:** 2013-03-02

**Authors:** Asma Lehleh, Mehdi Boutebdja, Adel Beghidja, Chahrazed Beghidja, Hocine Merazig

**Affiliations:** a Unité de Recherche de Chimie de l’Environnement et Moléculaire Structurale (CHEMS), Faculté des Sciences Exactes, Département de Chimie, Université de Constantine 1, 25000 Constantine, Algeria

## Abstract

The title compound, {[Co(C_10_H_8_N_2_)_2_(H_2_O)_2_](NO_3_)_2_·4H_2_O}_*n*_, (C_10_H_8_N_2_ = 4,4′-bipyridine = 4,4′-bpy) is a layered coordination polymer built up from a cationic square grid extending in (101) enclosing uncoordinating nitrate ions and water mol­ecules. The Co^II^ ion has site symmetry 2 and one of the 4,4′-bpy ligands is generated by twofold symmetry [two N atoms and two C atoms lie on the rotation axis and the dihedral angle between the pyridine rings is 45.66 (5)°]. The other 4,4′-bpy ligand is generated by a crystallographic inversion center. The Co^II^ ion exhibits a slightly distorted octa­hedral coordination geometry defined by two O atoms of two coordinating water mol­ecules and four N atoms from four bridging 4,4′-bpy ligands. The structure is consolidated by O—H⋯O, C—H⋯O and C—H⋯N hydrogen bonds.

## Related literature

For related structures with 4,4′-bpy ligands, see: Aoyagi *et al.* (2000 ▶); Felloni *et al.* (2002 ▶); Jin *et al.* (2006 ▶); Tong *et al.* (2000 ▶).
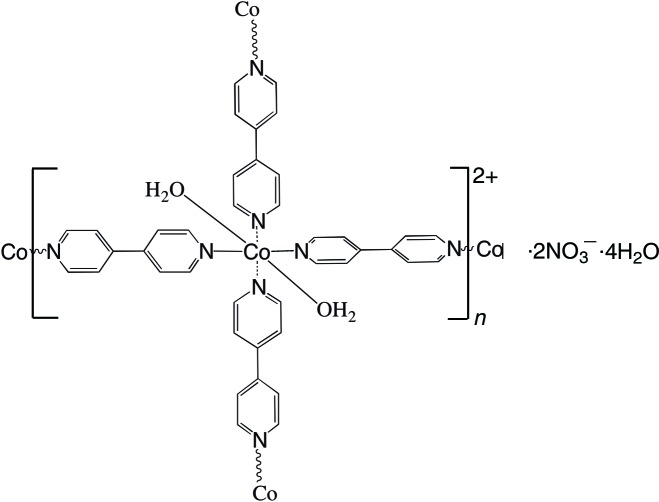


## Experimental

### 

#### Crystal data

[Co(C_10_H_8_N_2_)_2_(H_2_O)_2_](NO_3_)_2_·4H_2_O
*M* *_r_* = 603.41
Monoclinic, 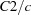

*a* = 18.6093 (19) Å
*b* = 11.5447 (13) Å
*c* = 12.1216 (13) Å
β = 95.625 (4)°
*V* = 2591.7 (5) Å^3^
*Z* = 4
Mo *K*α radiation
μ = 0.74 mm^−1^
*T* = 296 K
0.15 × 0.12 × 0.10 mm

#### Data collection

Bruker APEXII CCD diffractometer
18421 measured reflections
3942 independent reflections
3660 reflections with *I* > 2σ(*I*)
*R*_int_ = 0.016

#### Refinement

*R*[*F* ^2^ > 2σ(*F* ^2^)] = 0.030
*wR*(*F* ^2^) = 0.083
*S* = 1.05
3942 reflections
198 parameters
9 restraints
H atoms treated by a mixture of independent and constrained refinement
Δρ_max_ = 0.41 e Å^−3^
Δρ_min_ = −0.49 e Å^−3^

### 

Data collection: *APEX2* (Bruker, 2006 ▶); cell refinement: *SAINT* (Bruker, 2006 ▶); data reduction: *SAINT*; program(s) used to solve structure: *SHELXS97* (Sheldrick, 2008 ▶); program(s) used to refine structure: *SHELXL97* (Sheldrick, 2008 ▶); molecular graphics: *ATOMS* (Dowty, 1995 ▶); software used to prepare material for publication: *WinGX* (Farrugia, 2012 ▶).

## Supplementary Material

Crystal structure: contains datablock(s) global, I. DOI: 10.1107/S1600536813005230/hb7035sup1.cif

Structure factors: contains datablock(s) I. DOI: 10.1107/S1600536813005230/hb7035Isup2.hkl

Additional supplementary materials:  crystallographic information; 3D view; checkCIF report

## Figures and Tables

**Table 1:** Selected bond lengths (Å)

Co1—O1*W*	2.0741 (10)
Co1—N1	2.2235 (10)
Co1—N2	2.1898 (13)
Co1—N3^i^	2.2306 (14)

Symmetry code: (i) 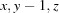.

**Table 2:** Hydrogen-bond geometry (Å, °)

*D*—H⋯*A*	*D*—H	H⋯*A*	*D*⋯*A*	*D*—H⋯*A*
O1*W*—H12⋯O3*W* ^ii^	0.877 (12)	1.801 (12)	2.675 (2)	174.2 (14)
O1*W*—H13⋯O2*W* ^iii^	0.884 (12)	1.790 (12)	2.6744 (18)	178.7 (16)
O2*W*—H14⋯O1	0.878 (13)	1.959 (12)	2.827 (2)	170.1 (13)
O2*W*—H15⋯O2^iv^	0.882 (14)	1.906 (14)	2.765 (2)	164.2 (14)
O3*W*—H16⋯O1	0.873 (13)	1.908 (13)	2.769 (3)	168.9 (14)
O3*W*—H17⋯O3^iv^	0.887 (11)	2.109 (11)	2.958 (2)	159.9 (15)
C1—H1⋯O1*W* ^v^	0.93	2.54	3.0860 (15)	117
C11—H11⋯N1^vi^	0.93	2.58	3.1974 (15)	124
C11—H11⋯O3^vii^	0.93	2.46	3.209 (2)	137

Symmetry codes: (ii) 
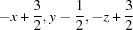
; (iii) 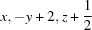; (iv) 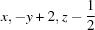; (v) 
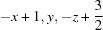
; (vi) 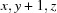; (vii) 
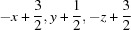
.

**Fig. 1.**
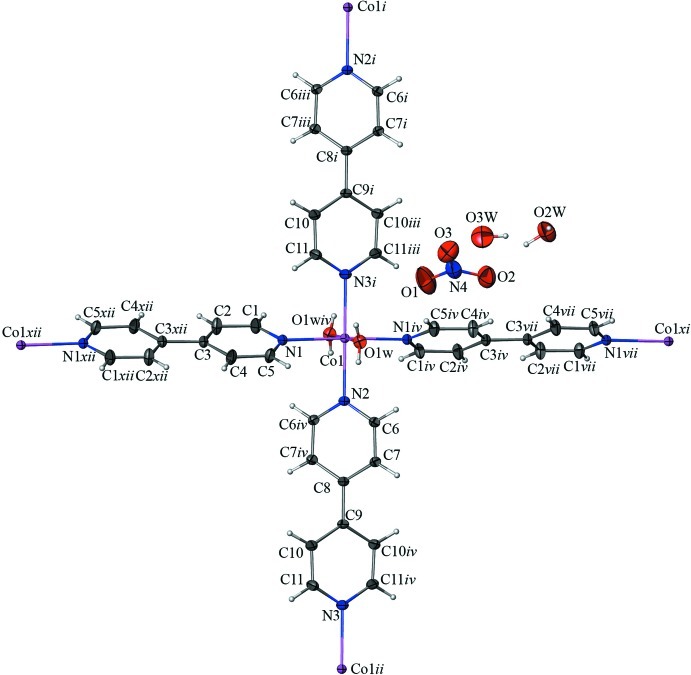
View of a fragment of the title compound with displacement ellipsoids for non-H atoms are drawn at the 50% probability level. symmetry code: i = x,-1 + y,z; ii = x,1 + y,z; iii = 1 - x,-1 + y,1/2 - z; iv = 1 - x,y,1/2 - z; vi = 3/2 - x,1/2 - y,-z; vii = 1/2 + x,-1/2 - y,-1/2 + z; xii = 3/2 - x,1/2 - y,1/2 - z

**Fig. 2.**
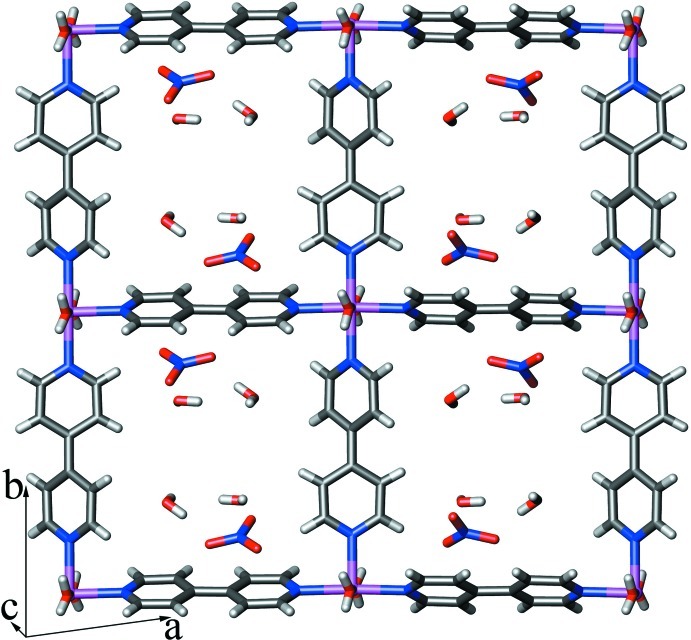
ATOMS view of the square grids of the title compound

**Fig. 3.**
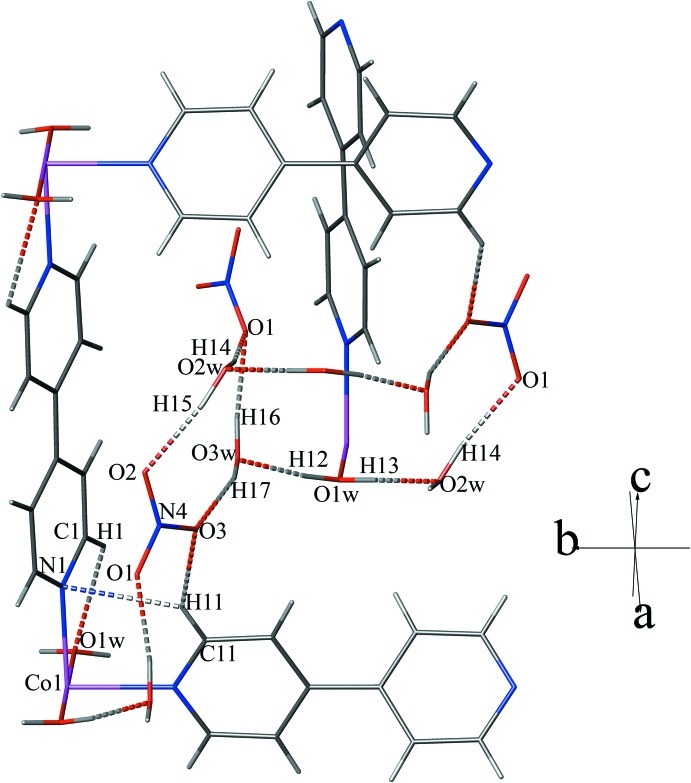
Partial view of the crystal structure of the title compound showing the hydrogen bonds

## supplementary crystallographic information

### Comment

The structure of (I) is a two dimensional layer with no interpenetration and no enclathration of organic guest molecules. The Co^II^ atom being located on a twofold crystallographic axis, has slightly distorted octahedral geometry, being ligated by two aqua ligands [Co—O1w = 2.074 (10) Å] and four pyridil groups from 4,4'-bpy ligands (Fig. 1), with a distance Co—N in the range of 2.1898 (13) - 2.2306 (14) Å which is almost similar to that observed in {[{Co(H_2_O)_2_}(µ-4,4'-bpy)_2_][NO_3_]_2_. 2(4,4'-bpy).2H_2_O}_n_; {[{Co(H_2_O)_2_(4,4'-bpy)_2_}(µ-4,4'-bpy)]. 1.5[NO_3_].0.5OH. 2(4,4'-bpy).2.5H_2_O}_n_, Felloni *et al.* (2002) and {[Co(µ-4,4'-bpy)(4,4'-bpy)_2_(H_2_O)_2_].(OH)_3_.(NMe_4_).4,4'-bpy.4H_2_O}_n_, Jin *et al.* (2006), these last act as a bidentate bridging ligands giving rise to a 2-D square grid sheet of (4,4) topology lying in the (101) plane. The basal coordinated 4,4'-bpy is located on an inversion center, it is planar with a 0° interplanar angle between the two pyridil rings, the same thing is observed in {[Cd(4,4'-bpy)_2_(H_2_O)_2_]. (NO_3_)_2_.4H_2_O}_n_, Aoyagi *et al.* (2000), whereas the axially one is nonplanar with an angle of 45.60° between the pyridil rings. The dihedral angle between the two basal coordinated 4,4'-bpy molecules is 46.92°. Each layer features a perfectly planar, though slightly distorted, square with Co(II) atom and 4,4'-bpy at each corner and side, respectively (*cis* N—Co—N = 88.64 (2)°, 91.37 (2)°) (Fig. 2). The square cavity has dimensions of 11.54 × 11.59 Å, which are comparable to those of closely related compound {[Cd(4,4'-bpy)_2_(H_2_O)_2_].(NO_3_)_2_.2H_2_O}_n_, Tong *et al.* (2000). The nitrate ion and lattice water molecules are situated between the coordination layers and form extensive hydrogen bonds among the aqua ligands, 4,4'-bpy, uncoordinated water molecules and nitrate ion, which extend the two-dimensional coordination layers into a three-dimensional molecular network (Fig. 3).

### Experimental

A mixture of Co(NO_3_)2. 6H_2_O (0.291 g, 1 mmole), *trans*-cinnamic acid (0.148 g, 1 mmole), NaOH (0.04 g, 1 mmole) and 4,4'-bpy (0.156 g, 1 mmole) were dissolved with 10 ml of mixed solution (MeOH/H_2_O: 2/1) in a 20 ml Teflon-lined stainless steel reactor and heated to 120°C for 24 h. The reactor was cooled to room temperature over a period of 24 h, the reaction was filtered. The orange filtrate was kept for several weeks at room temperature. Orange crystals suitable for X-ray analysis were obtained. Note: the measured crystal is a fragment cut from a larger crystal.

### Refinement

Water hydrogen atoms were tentatively found in the difference density Fourier map and were refined with an isotropic displacement parameter 1.5 that of the adjacent oxygen atom. The O—H distances were restrained to be 0.9 Å within a standard deviation of 0.01 with *U*_iso_(H) = 1.5 *U*_eq_(O) and the H···H contacts were restraint to 1.40 Å with a standard deviation of 0.02. A l l other Hydrogen atoms were placed in calculated positions with C —H distances of 0.93–0.96 Å for aromatic H atoms with *U*_iso_(H) =1.2 *U*_eq_(C).

### Crystal data

**Table:**

[Co(C_10_H_8_N_2_)_2_(H_2_O)_2_](NO_3_)_2_·4H_2_O	*Z* = 4
*M**_r_* = 603.41	*F*(000) = 1252
Monoclinic, *C*2/*c*	Least Squares Treatment of 25 SET4 setting angles.
Hall symbol: -C 2yc	*D*_x_ = 1.546 Mg m^−^^3^
*a* = 18.6093 (19) Å	Mo *K*α radiation, λ = 0.71073 Å
*b* = 11.5447 (13) Å	µ = 0.74 mm^−^^1^
*c* = 12.1216 (13) Å	*T* = 296 K
β = 95.625 (4)°	Block, orange
*V* = 2591.7 (5) Å^3^	0.15 × 0.12 × 0.10 mm

### Data collection

**Table:**

Bruker APEXII CCD diffractometer	3660 reflections with *I* > 2σ(*I*)
Radiation source: sealed tube	*R*_int_ = 0.016
Graphite monochromator	θ_max_ = 30.5°, θ_min_ = 3.7°
Detector resolution: 18.4 pixels mm^-1^	*h* = −26→26
φ and ω scans	*k* = −16→12
18421 measured reflections	*l* = −15→17
3942 independent reflections	

### Refinement

**Table:**

Refinement on *F*^2^	Secondary atom site location: difference Fourier map
Least-squares matrix: full	Hydrogen site location: inferred from neighbouring sites
*R*[*F*^2^ > 2σ(*F*^2^)] = 0.030	H atoms treated by a mixture of independent and constrained refinement
*wR*(*F*^2^) = 0.083	*w* = 1/[σ^2^(*F*_o_^2^) + (0.0411*P*)^2^ + 2.4609*P*] where *P* = (*F*_o_^2^ + 2*F*_c_^2^)/3
*S* = 1.05	(Δ/σ)_max_ = 0.001
3942 reflections	Δρ_max_ = 0.41 e Å^−^^3^
198 parameters	Δρ_min_ = −0.49 e Å^−^^3^
9 restraints	Extinction correction: *SHELXL97* (Sheldrick, 2008), FC^*^=KFC[1+0.001XFC^2^Λ^3^/SIN(2Θ)]^-1/4^
Primary atom site location: structure-invariant direct methods	Extinction coefficient: 0.0058 (4)

### Special details

**Table:**

Geometry. Bond distances, angles *etc*. have been calculated using the rounded fractional coordinates. All su's are estimated from the variances of the (full) variance-covariance matrix. The cell e.s.d.'s are taken into account in the estimation of distances, angles and torsion angles
Refinement. Refinement on *F*^2^ for ALL reflections except those flagged by the user for potential systematic errors. Weighted *R*-factors *wR* and all goodnesses of fit *S* are based on *F*^2^, conventional *R*-factors *R* are based on *F*, with *F* set to zero for negative *F*^2^. The observed criterion of *F*^2^ > σ(*F*^2^) is used only for calculating -*R*-factor-obs *etc*. and is not relevant to the choice of reflections for refinement. *R*-factors based on *F*^2^ are statistically about twice as large as those based on *F*, and *R*-factors based on ALL data will be even larger.

### Fractional atomic coordinates and isotropic or equivalent isotropic displacement parameters (Å^2^)

**Table:**

	*x*	*y*	*z*	*U*_iso_*/*U*_eq_	
Co1	0.50000	0.74238 (2)	0.75000	0.0160 (1)	
O1W	0.56526 (5)	0.74192 (8)	0.89887 (8)	0.0261 (3)	
N1	0.59538 (5)	0.74697 (8)	0.65303 (8)	0.0194 (3)	
N2	0.50000	0.93206 (11)	0.75000	0.0198 (3)	
N3	0.50000	1.54917 (12)	0.75000	0.0216 (4)	
C1	0.59232 (6)	0.70962 (12)	0.54799 (10)	0.0261 (3)	
C2	0.65075 (7)	0.71019 (13)	0.48560 (10)	0.0286 (3)	
C3	0.71766 (6)	0.75068 (9)	0.53140 (10)	0.0196 (3)	
C4	0.72032 (7)	0.79164 (13)	0.63952 (11)	0.0289 (3)	
C5	0.65925 (7)	0.78805 (12)	0.69653 (11)	0.0282 (3)	
C6	0.48157 (7)	0.99302 (10)	0.83731 (10)	0.0229 (3)	
C7	0.48067 (7)	1.11296 (10)	0.84048 (10)	0.0237 (3)	
C8	0.50000	1.17564 (13)	0.75000	0.0189 (4)	
C9	0.50000	1.30435 (13)	0.75000	0.0191 (4)	
C10	0.55676 (7)	1.36752 (10)	0.71325 (11)	0.0266 (3)	
C11	0.55528 (7)	1.48777 (10)	0.71687 (12)	0.0277 (3)	
O1	0.73251 (11)	1.0878 (2)	0.67235 (12)	0.0843 (7)	
O2	0.67234 (8)	1.07423 (15)	0.81345 (14)	0.0629 (5)	
O3	0.77569 (8)	0.99156 (14)	0.81568 (13)	0.0614 (5)	
N4	0.72726 (8)	1.05039 (14)	0.76769 (11)	0.0436 (4)	
O2W	0.63340 (8)	1.06829 (14)	0.48099 (11)	0.0546 (4)	
O3W	0.84438 (9)	1.06853 (16)	0.53876 (15)	0.0701 (6)	
H1	0.54840	0.68170	0.51520	0.0310*	
H2	0.64530	0.68350	0.41290	0.0340*	
H4	0.76320	0.82160	0.67380	0.0350*	
H5	0.66290	0.81570	0.76890	0.0340*	
H6	0.46880	0.95260	0.89880	0.0280*	
H7	0.46720	1.15130	0.90270	0.0280*	
H10	0.59550	1.32940	0.68640	0.0320*	
H11	0.59480	1.52800	0.69510	0.0330*	
H12	0.5930 (7)	0.6840 (9)	0.9229 (13)	0.0240*	
H13	0.5878 (8)	0.8051 (9)	0.9250 (13)	0.0240*	
H14	0.6676 (7)	1.0786 (14)	0.5353 (10)	0.0240*	
H15	0.6542 (8)	1.0272 (13)	0.4316 (10)	0.0240*	
H16	0.8124 (7)	1.0682 (15)	0.5868 (10)	0.0240*	
H17	0.8192 (8)	1.0679 (15)	0.4727 (8)	0.0240*	

### Atomic displacement parameters (Å^2^)

**Table:**

	*U*^11^	*U*^22^	*U*^33^	*U*^12^	*U*^13^	*U*^23^
Co1	0.0164 (1)	0.0132 (1)	0.0193 (1)	0.0000	0.0057 (1)	0.0000
O1W	0.0236 (4)	0.0290 (5)	0.0255 (4)	0.0002 (3)	0.0014 (3)	0.0015 (3)
N1	0.0188 (4)	0.0167 (4)	0.0240 (5)	−0.0001 (3)	0.0084 (3)	−0.0002 (3)
N2	0.0233 (6)	0.0136 (5)	0.0232 (6)	0.0000	0.0060 (5)	0.0000
N3	0.0222 (6)	0.0148 (6)	0.0282 (7)	0.0000	0.0046 (5)	0.0000
C1	0.0183 (5)	0.0369 (6)	0.0238 (5)	−0.0031 (4)	0.0053 (4)	−0.0024 (5)
C2	0.0208 (5)	0.0456 (7)	0.0203 (5)	−0.0038 (5)	0.0062 (4)	−0.0054 (5)
C3	0.0175 (5)	0.0210 (5)	0.0215 (5)	−0.0001 (4)	0.0075 (4)	−0.0002 (4)
C4	0.0213 (5)	0.0377 (7)	0.0291 (6)	−0.0080 (5)	0.0095 (4)	−0.0131 (5)
C5	0.0246 (5)	0.0341 (6)	0.0278 (6)	−0.0069 (5)	0.0116 (4)	−0.0125 (5)
C6	0.0321 (6)	0.0160 (5)	0.0219 (5)	−0.0013 (4)	0.0090 (4)	0.0009 (4)
C7	0.0344 (6)	0.0160 (5)	0.0220 (5)	−0.0016 (4)	0.0099 (4)	−0.0022 (4)
C8	0.0210 (6)	0.0135 (6)	0.0227 (7)	0.0000	0.0042 (5)	0.0000
C9	0.0232 (7)	0.0132 (6)	0.0211 (6)	0.0000	0.0032 (5)	0.0000
C10	0.0257 (5)	0.0161 (5)	0.0401 (7)	0.0027 (4)	0.0136 (5)	0.0021 (4)
C11	0.0256 (5)	0.0168 (5)	0.0428 (7)	−0.0001 (4)	0.0133 (5)	0.0036 (5)
O1	0.0952 (13)	0.1202 (16)	0.0372 (7)	−0.0386 (12)	0.0056 (7)	0.0049 (9)
O2	0.0435 (7)	0.0741 (10)	0.0729 (10)	−0.0014 (7)	0.0144 (6)	0.0011 (8)
O3	0.0455 (7)	0.0733 (10)	0.0632 (9)	0.0023 (7)	−0.0057 (6)	−0.0158 (8)
N4	0.0410 (7)	0.0553 (8)	0.0341 (6)	−0.0193 (6)	0.0017 (5)	−0.0078 (6)
O2W	0.0554 (8)	0.0676 (9)	0.0402 (6)	0.0282 (7)	0.0015 (5)	0.0039 (6)
O3W	0.0645 (10)	0.0731 (11)	0.0726 (11)	−0.0275 (8)	0.0065 (8)	−0.0237 (9)

### Geometric parameters (Å, º)

**Table:**

Co1—O1W	2.0741 (10)	N3—C11	1.3428 (15)
Co1—N1	2.2235 (10)	C1—C2	1.3840 (17)
Co1—N2	2.1898 (13)	C2—C3	1.3934 (17)
Co1—N3^i^	2.2306 (14)	C3—C4	1.3896 (18)
Co1—O1W^ii^	2.0741 (10)	C3—C3^iii^	1.4860 (16)
Co1—N1^ii^	2.2235 (10)	C4—C5	1.3874 (19)
O1W—H13	0.884 (12)	C6—C7	1.3854 (16)
O1W—H12	0.877 (12)	C7—C8	1.3904 (14)
O1—N4	1.247 (2)	C8—C9	1.486 (2)
O2—N4	1.241 (2)	C9—C10^ii^	1.3922 (15)
O3—N4	1.229 (2)	C9—C10	1.3922 (15)
O2W—H14	0.878 (13)	C10—C11	1.3893 (16)
O2W—H15	0.882 (14)	C1—H1	0.9300
O3W—H17	0.887 (11)	C2—H2	0.9300
O3W—H16	0.873 (13)	C4—H4	0.9300
N1—C1	1.3402 (16)	C5—H5	0.9300
N1—C5	1.3390 (16)	C6—H6	0.9300
N2—C6^ii^	1.3432 (14)	C7—H7	0.9300
N2—C6	1.3432 (14)	C10—H10	0.9300
N3—C11^ii^	1.3428 (15)	C11—H11	0.9300
			
O1W—Co1—N1	91.74 (4)	C1—C2—C3	120.21 (11)
O1W—Co1—N2	90.15 (3)	C2—C3—C4	115.96 (11)
O1W—Co1—N3^i^	89.85 (3)	C2—C3—C3^iii^	121.97 (11)
O1W—Co1—O1W^ii^	179.71 (4)	C3^iii^—C3—C4	122.07 (11)
O1W—Co1—N1^ii^	88.27 (4)	C3—C4—C5	120.29 (12)
N1—Co1—N2	88.64 (2)	N1—C5—C4	123.59 (12)
N1—Co1—N3^i^	91.37 (2)	N2—C6—C7	123.39 (11)
O1W^ii^—Co1—N1	88.27 (4)	C6—C7—C8	119.57 (11)
N1—Co1—N1^ii^	177.27 (4)	C7—C8—C9	121.36 (7)
N2—Co1—N3^i^	180.00	C7^ii^—C8—C9	121.36 (7)
O1W^ii^—Co1—N2	90.15 (3)	C7—C8—C7^ii^	117.28 (13)
N1^ii^—Co1—N2	88.64 (2)	C8—C9—C10^ii^	121.59 (7)
O1W^ii^—Co1—N3^i^	89.85 (3)	C8—C9—C10	121.59 (7)
N1^ii^—Co1—N3^i^	91.37 (2)	C10—C9—C10^ii^	116.82 (13)
O1W^ii^—Co1—N1^ii^	91.74 (4)	C9—C10—C11	119.69 (12)
H12—O1W—H13	105.5 (12)	N3—C11—C10	123.72 (12)
Co1—O1W—H12	124.5 (9)	N1—C1—H1	118.00
Co1—O1W—H13	121.8 (9)	C2—C1—H1	118.00
H14—O2W—H15	104.3 (13)	C1—C2—H2	120.00
H16—O3W—H17	105.5 (12)	C3—C2—H2	120.00
Co1—N1—C5	121.61 (8)	C5—C4—H4	120.00
Co1—N1—C1	122.19 (7)	C3—C4—H4	120.00
C1—N1—C5	116.20 (10)	C4—C5—H5	118.00
Co1—N2—C6^ii^	121.60 (7)	N1—C5—H5	118.00
C6—N2—C6^ii^	116.81 (12)	N2—C6—H6	118.00
Co1—N2—C6	121.60 (7)	C7—C6—H6	118.00
C11—N3—C11^ii^	116.27 (12)	C6—C7—H7	120.00
Co1^iv^—N3—C11^ii^	121.86 (7)	C8—C7—H7	120.00
Co1^iv^—N3—C11	121.86 (7)	C11—C10—H10	120.00
O1—N4—O2	118.72 (17)	C9—C10—H10	120.00
O2—N4—O3	120.61 (15)	C10—C11—H11	118.00
O1—N4—O3	120.67 (17)	N3—C11—H11	118.00
N1—C1—C2	123.72 (11)		
			
O1W—Co1—N1—C1	156.61 (10)	C11^ii^—N3—C11—C10	1.48 (17)
N2—Co1—N1—C1	−113.29 (9)	N1—C1—C2—C3	0.3 (2)
N3^i^—Co1—N1—C1	66.72 (9)	C1—C2—C3—C4	−1.58 (19)
O1W^ii^—Co1—N1—C1	−23.10 (10)	C1—C2—C3—C3^iii^	178.01 (12)
O1W—Co1—N1—C5	−23.77 (10)	C4—C3—C3^iii^—C2^iii^	−0.44 (18)
N2—Co1—N1—C5	66.34 (9)	C2—C3—C4—C5	1.63 (19)
N3^i^—Co1—N1—C5	−113.66 (9)	C3^iii^—C3—C4—C5	−177.96 (12)
O1W^ii^—Co1—N1—C5	156.53 (10)	C2—C3—C3^iii^—C2^iii^	−179.98 (15)
O1W—Co1—N2—C6	−53.00 (7)	C2—C3—C3^iii^—C4^iii^	0.44 (18)
N1—Co1—N2—C6	−144.74 (7)	C4—C3—C3^iii^—C4^iii^	−179.98 (14)
O1W^ii^—Co1—N2—C6	127.00 (7)	C3—C4—C5—N1	−0.4 (2)
N1^ii^—Co1—N2—C6	35.27 (7)	N2—C6—C7—C8	−0.39 (18)
O1W—Co1—N2—C6^ii^	127.00 (7)	C6—C7—C8—C9	−179.82 (9)
N1—Co1—N2—C6^ii^	35.27 (7)	C6—C7—C8—C7^ii^	0.18 (15)
Co1—N1—C1—C2	−179.36 (10)	C7—C8—C9—C10^ii^	−44.87 (9)
C5—N1—C1—C2	1.00 (19)	C7^ii^—C8—C9—C10	−44.87 (9)
Co1—N1—C5—C4	179.41 (11)	C7—C8—C9—C10	135.13 (9)
C1—N1—C5—C4	−0.95 (19)	C8—C9—C10—C11	−178.64 (9)
Co1—N2—C6—C7	−179.80 (9)	C10^ii^—C9—C10—C11	1.36 (15)
C6^ii^—N2—C6—C7	0.20 (15)	C9—C10—C11—N3	−2.9 (2)
Co1^iv^—N3—C11—C10	−178.53 (10)		

Symmetry codes: (i) *x*, *y*−1, *z*; (ii) −*x*+1, *y*, −*z*+3/2; (iii) −*x*+3/2, −*y*+3/2, −*z*+1; (iv) *x*, *y*+1, *z*.

### Hydrogen-bond geometry (Å, º)

**Table:**

*D*—H···*A*	*D*—H	H···*A*	*D*···*A*	*D*—H···*A*
O1*W*—H12···O3*W*^v^	0.877 (12)	1.801 (12)	2.675 (2)	174.2 (14)
O1*W*—H13···O2*W*^vi^	0.884 (12)	1.790 (12)	2.6744 (18)	178.7 (16)
O2*W*—H14···O1	0.878 (13)	1.959 (12)	2.827 (2)	170.1 (13)
O2*W*—H15···O2^vii^	0.882 (14)	1.906 (14)	2.765 (2)	164.2 (14)
O3*W*—H16···O1	0.873 (13)	1.908 (13)	2.769 (3)	168.9 (14)
O3*W*—H17···O3^vii^	0.887 (11)	2.109 (11)	2.958 (2)	159.9 (15)
C1—H1···O1*W*^ii^	0.93	2.54	3.0860 (15)	117
C11—H11···N1^iv^	0.93	2.58	3.1974 (15)	124
C11—H11···O3^viii^	0.93	2.46	3.209 (2)	137

Symmetry codes: (ii) −*x*+1, *y*, −*z*+3/2; (iv) *x*, *y*+1, *z*; (v) −*x*+3/2, *y*−1/2, −*z*+3/2; (vi) *x*, −*y*+2, *z*+1/2; (vii) *x*, −*y*+2, *z*−1/2; (viii) −*x*+3/2, *y*+1/2, −*z*+3/2.
